# Supplementary material for: Divergent receptor proteins confer responses to different karrikins in two ephemeral weeds
Source: Nat Commun. 2020 Mar 9;11:1264. doi: 10.1038/s41467-020-14991-w (PMC7062792; doi:10.1038/s41467-020-14991-w)
Supplement: Supplementary file 1 — Supplementary Information [file 41467_2020_14991_MOESM1_ESM.pdf]

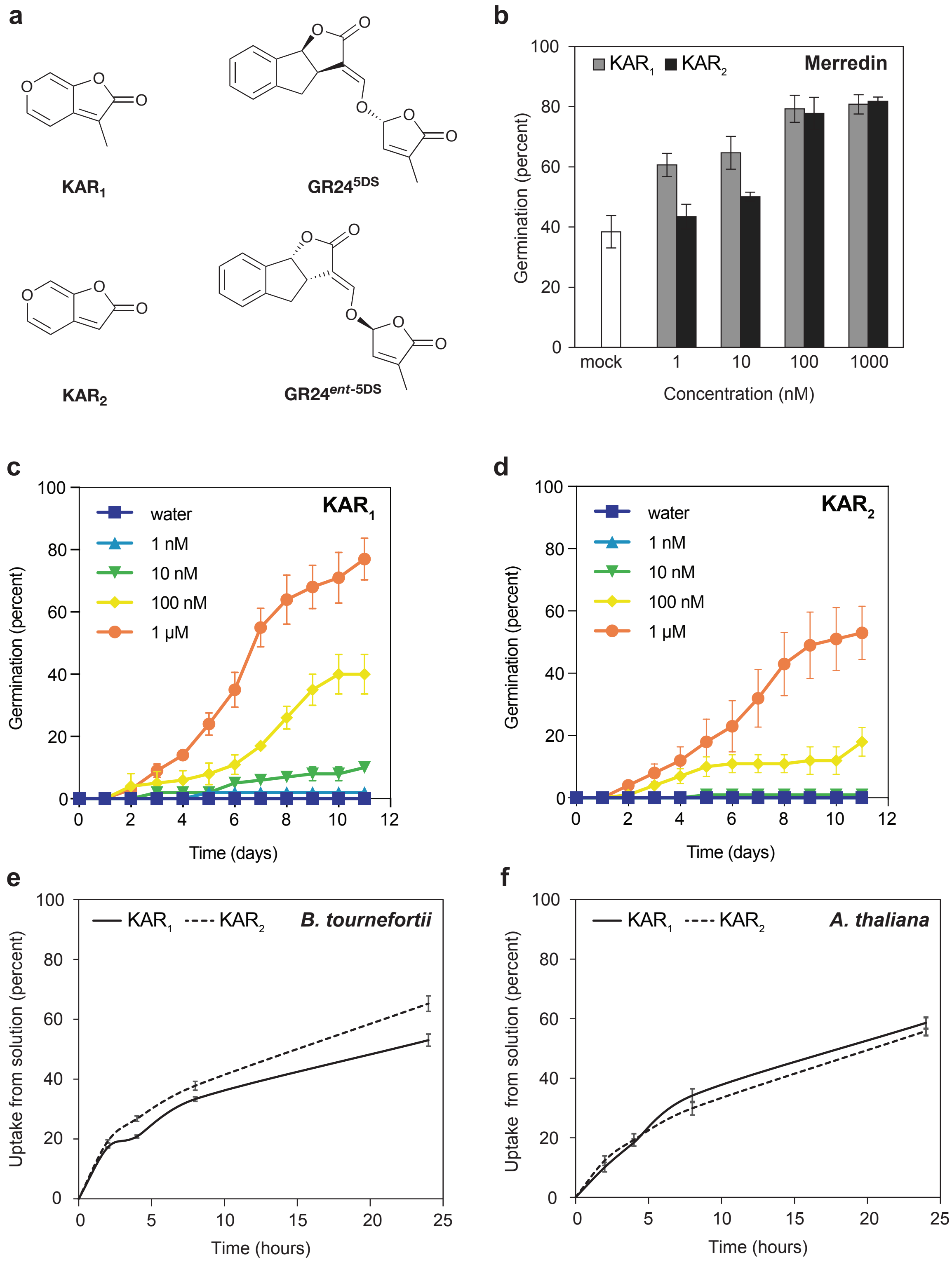

Supplementary Figure 1. Germination of *Brassica tournefortii* seeds treated with karrikins
a, Structures of KAR_1, KAR_2 and two enantiomers of GR24. **b,** Germination response of the "Merridin" batch of *B. tournefortii* seed treated with KAR_1 and KAR_2 after three days. **c-d,** Germination response of the "Perth" batch to KAR_1 (**c**) and KAR_2 (**d**). Data in Figure 1a are derived from the data shown in **c** and **d.** **e-f**, Rates of KAR_1 and KAR_2 uptake by imbibed seed, as determined by GC-MS. All error bars are mean ± SE of n = 3 batches of 75 seed (b-d) or 3 samples of 40 mg (e) or 20 mg (f) seed as described in Methods. Source data are provided as a Source Data file.

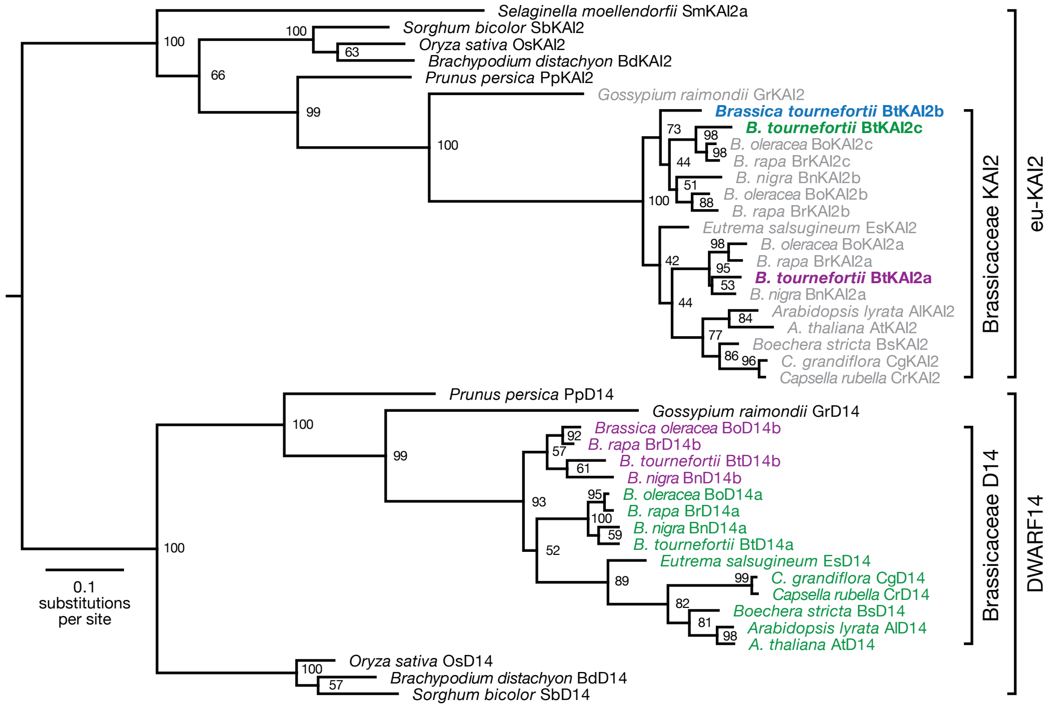

Supplementary Figure 2. Extended phylogeny of eu-KAI2 and D14 proteins in angiosperms
Maximum likelihood phylogeny of KAI2 and D14 homologues in the Brassicaceae and monocots, based on nucleotide data. Node values represent bootstrap support from 100 replicates. A KAI2 sequence from *Selaginella moellendorffii* (SmKAI2a) serves as an outgroup for the eu-KAI2 clade. Source data are provided as a Source Data file.

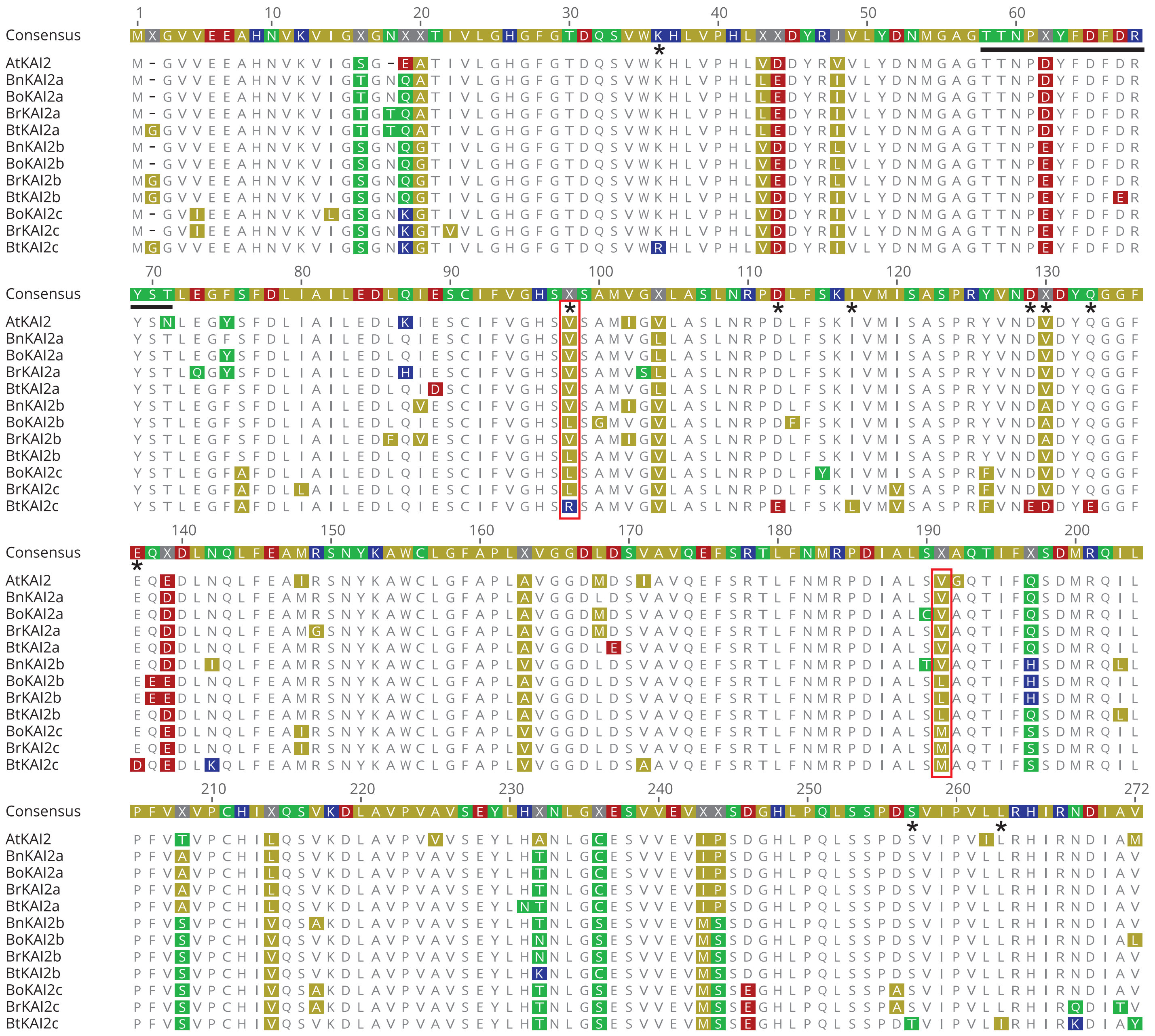

Supplementary Figure 3. Alignment of *Brassica* KAI2 sequences
Full length protein coding regions of KAI2 homologues from four *Brassica* species (*Brassica tournefortii*, *B. rapa*, *B. nigra* and *B. oleracea*) were translated from database nucleic acid sequences and aligned to Arabidopsis KAI2 using MAFFT (ref. 1) implemented in Geneious R10 software (Biomatters Ltd). Amino acid residues are coloured according to polarity: yellow, non-polar (G, A, V, L, I, F, W, M, P); green, polar & uncharged (S, T, C, Y, N, Q); red, polar & acidic (D, E); blue, polar & basic (K, R, H). Residues that are unique to BtKAI2c but otherwise invariant are highlighted with asterisks (*); residues 98 and 191 are highlighted with red boxes. The underlined region indicates the epitope used to raise the antibody against AtKAI2. Source data are provided as a Source Data file.

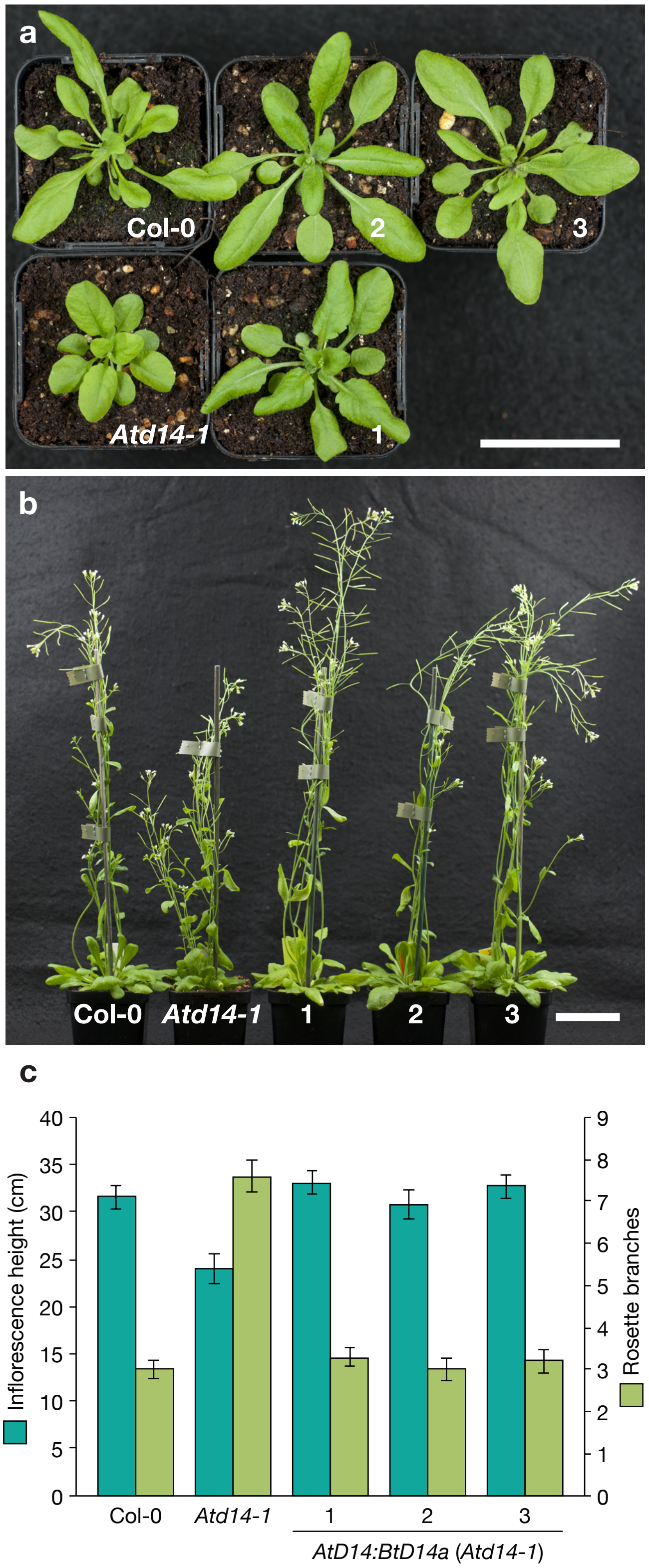

Supplementary Figure 4. BtD14a is functionally homologous to AtD14
Three independent transgenic lines of Arabidopsis *Atd14-1* were analysed for functional complementation of the mutant phenotype by an *AtD14pro:BtD14a* transgene. **a**, Rosette and leaf morphology at 31 days post-germination. **b**, Plant height and number of primary rosette branches at 45 days post-germination. **c**, Quantification of height and branching parameters, means ± SE, *n* = 10 plants per genotype. Scale bars: 50 mm. Source data are provided as a Source Data file.

a

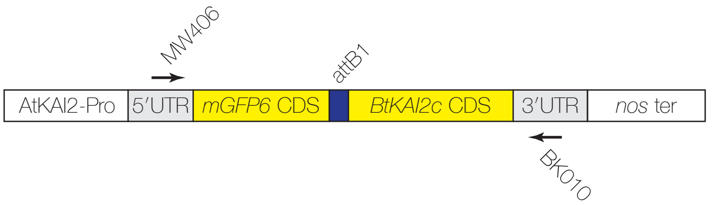

b

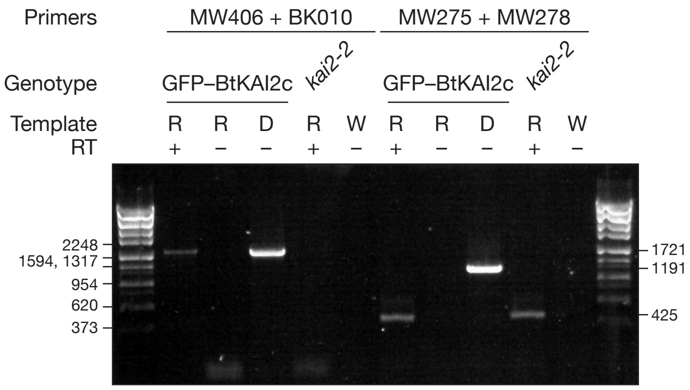

Primers	cDNA (R)	gDNA (D)
MW406 + BK010	1721	1721
MW275 + MW278	425	1191

c

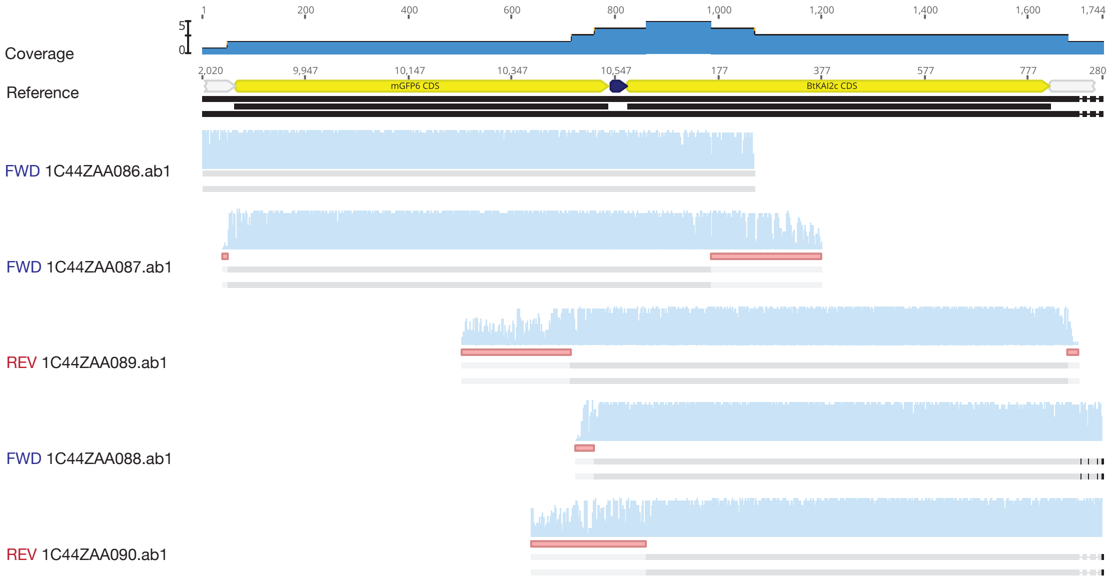

d

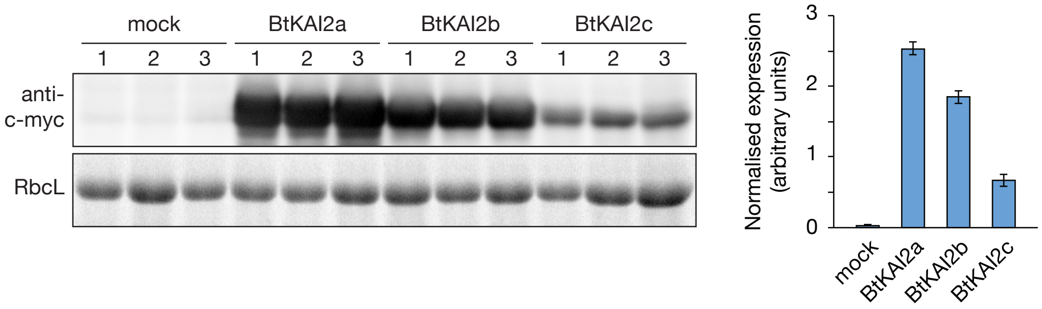

Supplementary Figure 5. The *GFP-BtKAI2c* transgene is faithfully transcribed in Arabidopsis

a, Structure of the *AtKAI2pro:mGFP6-BtKAI2c* transgene. Primers used for RT-PCR are shown with arrows. The promoter and 5′UTR are derived from *At4g37470* (*AtKAI2*). attB1, Gateway recombination site that links mGFP6 and BtKAI2c regions; *nos* ter, nopaline synthase terminator. Not drawn to scale.

b, RT-PCR analysis of *GFP-BtKAI2c* transcripts after 35 cycles of amplification. Primers MW406 + BK010 target the transgene, while a second primer pair (MW275 + MW278) serves as a control and spans five introns of *At1g03055*. *kai2-2* serves as a non-transgenic control genotype. Templates: R, total RNA; D, genomic DNA; W, water only. RT, reverse transcriptase (Supercript III). DNA size standards (in base pairs) are indicated on the left, with anticipated PCR product sizes shown on the right and defined in the table.

c, The RT-PCR product generated with proof-reading polymerase (Q5, New England Biolabs) and primers MW406 and BK010 was cloned into pCR4-TOPO (Life Technologies). Five dideoxy sequence traces were aligned against the *GFP-BtKAI2c* transgene reference. No disagreements with the reference sequence were observed. Red bars indicate trimmed regions of sequence traces to remove low quality data.

d, Transient expression of BtKAI2a, BtKAI2b and BtKAI2c proteins in tobacco. Plasmids encoding N-terminal, c-myc-tagged proteins were transferred to Agrobacterium, and the resulting strains used to infiltrate tobacco leaves. After 96 h, samples were harvested in triplicate (two to three leaves per sample). Mock-treated leaves were transformed with a plasmid encoding a non-tagged protein. Sixty micrograms of total protein were separated by SDS-PAGE, blotted and challenged with anti-c-myc antibody (Genscript A00704). Band intensity was measured using ImageJ, and expression was normalised to intensity of the Rubisco large subunit (RbcL) band on the “stain-free” gel imaged under UV light. Error bars indicate SE, n = 3 replicates. Source data are provided as a Source Data file.

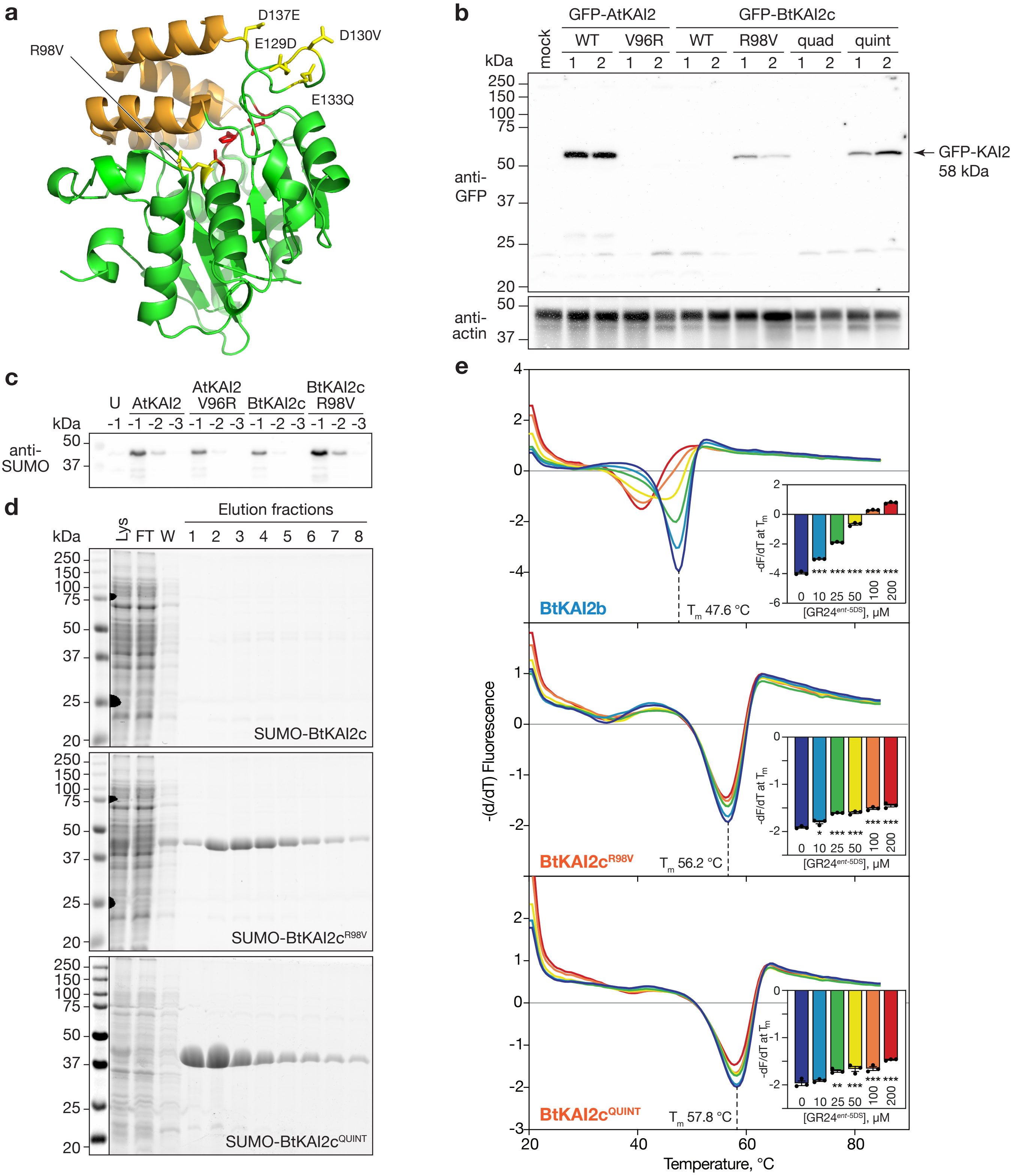

Supplementary Figure 6. The R98V mutation restores stability of BtKAI2c

a, Homology model of the native BtKAI2c protein. Highlighted in yellow are five residues and their corresponding mutations that were assessed for effects on BtKAI2c stability. The combination of E129D, D130V, E133Q and D173E in the hinge region between the lid domain (orange) and core domain (green) is the quadruple ("quad") mutant; combining this with R98V yields the quintuple ("quint") mutant. Red residues are the Ser-His-Asp catalytic triad. **b**, Transient expression of AtKAI2 and BtKAI2c variants in tobacco leaves following infiltration by Agrobacterium strains encoding GFP-KAI2 variants driven by the CaMV *35S* promoter. Three leaves were infiltrated on each of two different plants. After five days, 20 μg soluble protein was electrophoresed, blotted and challenged with antibodies against either anti-GFP (top) or anti-actin (bottom). **c**, Immunoblot of crude bacterial lysates expressing SUMO fusion proteins and challenged with anti-SUMO antibody. Negative values above each lane indicate $\log_{10}$ dilution factors of lysate; U, lysate from uninduced bacterial culture expressing SUMO-AtKAI2.**d**, SDS-PAGE analysis of protein purification runs of SUMO-BtKAI2c, -BtKAI2c^{R98V} and -BtKAI2c^{QUINT} harvested from 900 mL bacterial culture. Lys, clarified lysate; FT, column flow through after immobilisation on Co^{2+} affinity column; W, wash with 10 mM imidazole. Proteins were eluted with 200 mM imidazole in eight successive fractions. **e**, DSF curves of SUMO fusion proteins treated with 0-200 μM GR24$^{ent\text{-}5DS}$. Insets plot the minimum value of –(dF/dT) at the melting point of the protein as determined in the absence of ligand (means ± SE, n = 3). Significant differences from untreated control: * $P < 0.05$ ** $P < 0.01$ *** $P < 0.001$ (ANOVA). Source data are provided as a Source Data file.

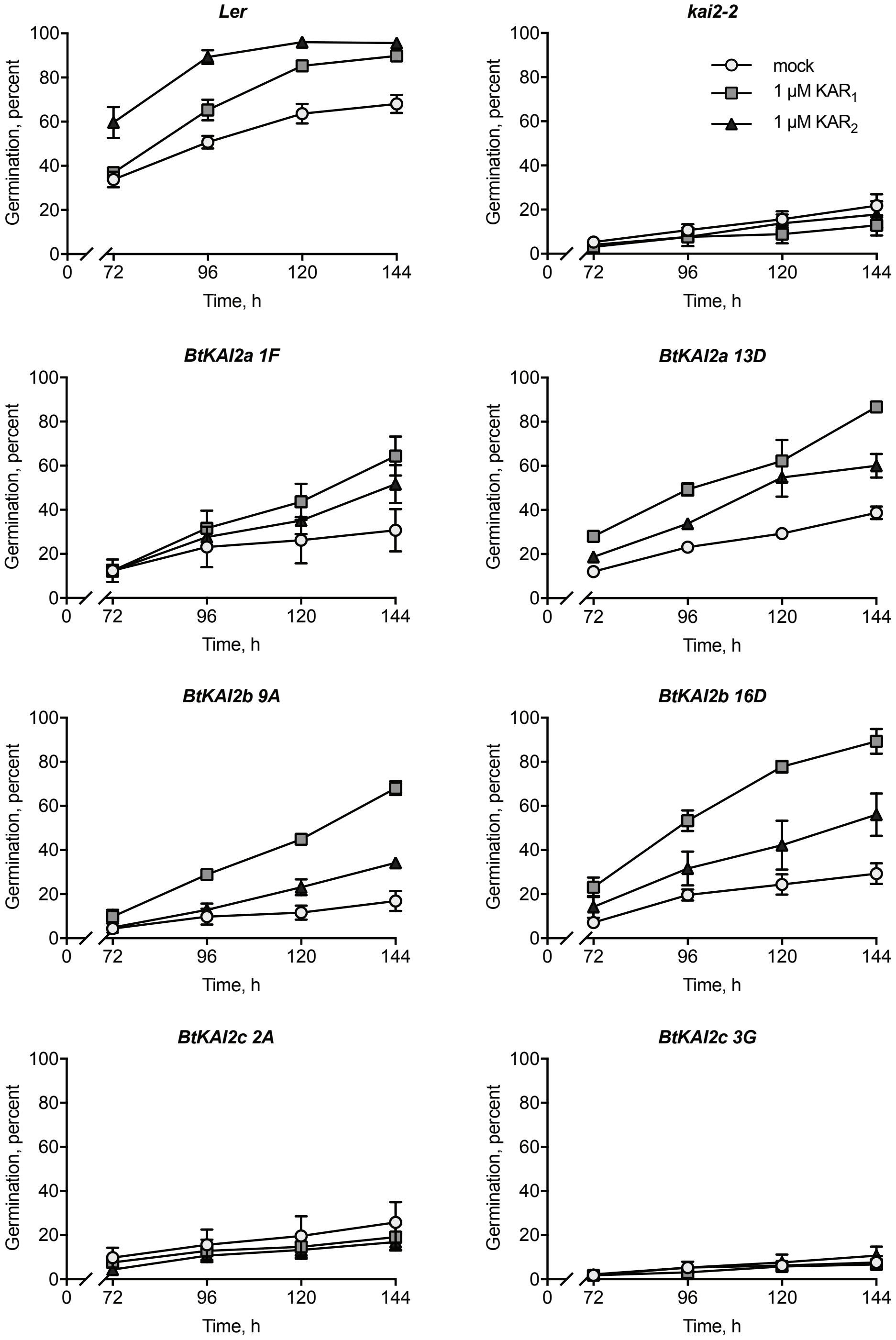

Supplementary Figure 7. Germination profiles of transgenic Arabidopsis seeds expressing native BtKAI2 homologues

Freshly harvested seed (three batches per genotype, each batch harvested from four plants) were removed from freezer storage, surface-sterilised and sown on 1% Phytagel supplemented with 0.1% acetone (mock), 1 μM KAR_1 or 1 μM KAR_2. Seed were incubated under constant light at 25 °C. Seed were examined for germination (radicle protrusion) 72 h after sowing and every 24 h thereafter. Data are means ± SE of three independent seed batches and 75 seed per batch. For each transgene, two independent, homozygous transgenic lines were analysed. Data presented in Figure 3 of the main manuscript are derived from these data. Source data are provided as a Source Data file.

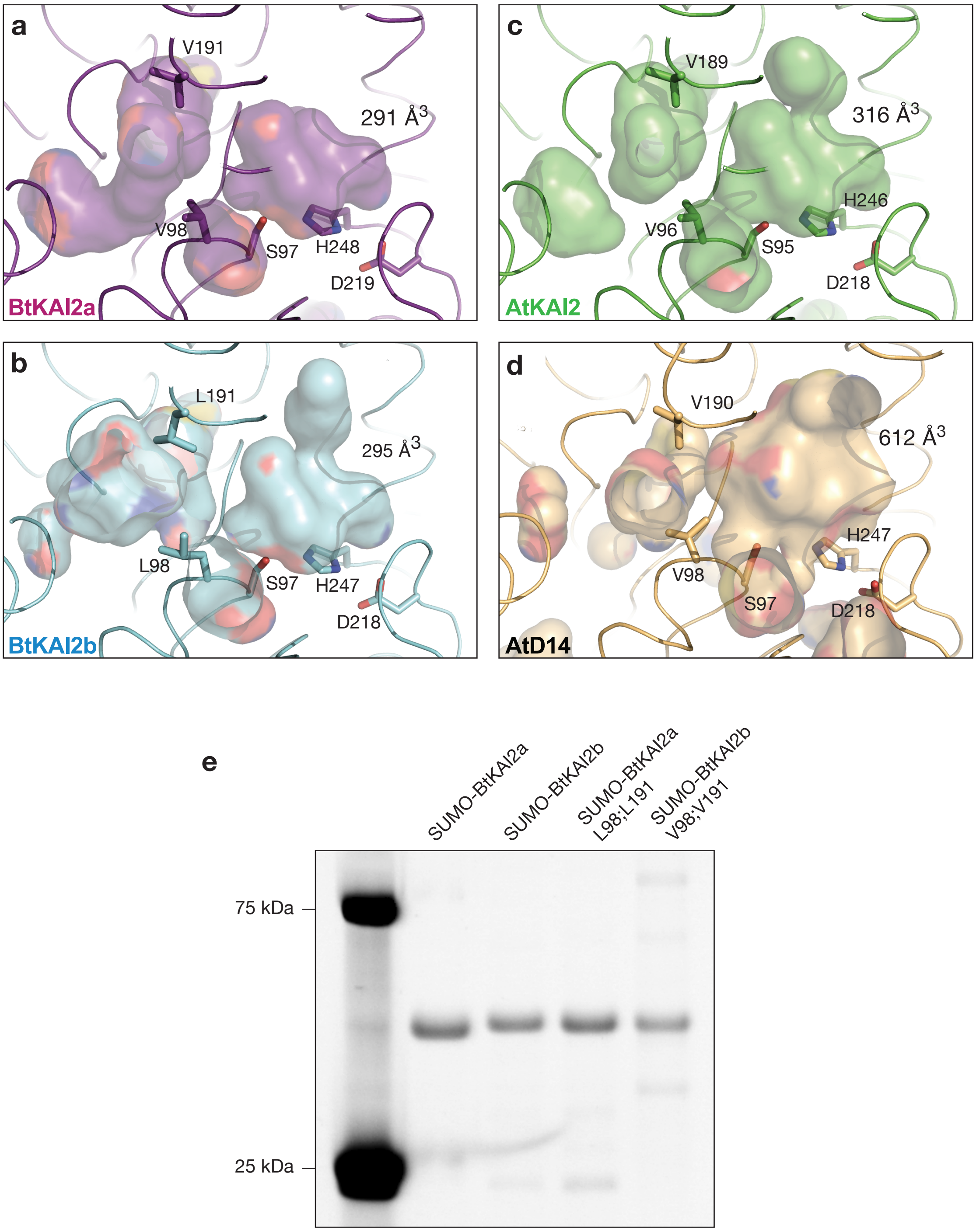

Supplementary Figure 8. BtKAI2 homology models and SDS-PAGE of SUMO-BtKAI2 fusion proteins used for DSF.
a-d, Solved structure of AtKAI2 (PDB: 3w06, ref. 2), AtD14 (PDB: 4IH4, ref. 3) and predicted homology models of BtKAI2a and BtKAI2b. Coloured surfaces depict internal cavities; values indicate the volumes of the primary ligand-binding cavities, adjacent to the catalytic Ser-His-Asp residues. Also shown is a variable secondary pocket, to the left of the primary pocket in these images.
e, To assess purity after affinity chromatography, five micrograms of each purified protein was electrophoresed on a 12% acrylamide gel containing 2,2,2-trichloroethanol and visualised under UV light. Protein size standards at 75 and 25 kDa (Bio-Rad Precision Plus Dual Colour) fluoresce strongly under UV light. Source data are provided as a Source Data file.

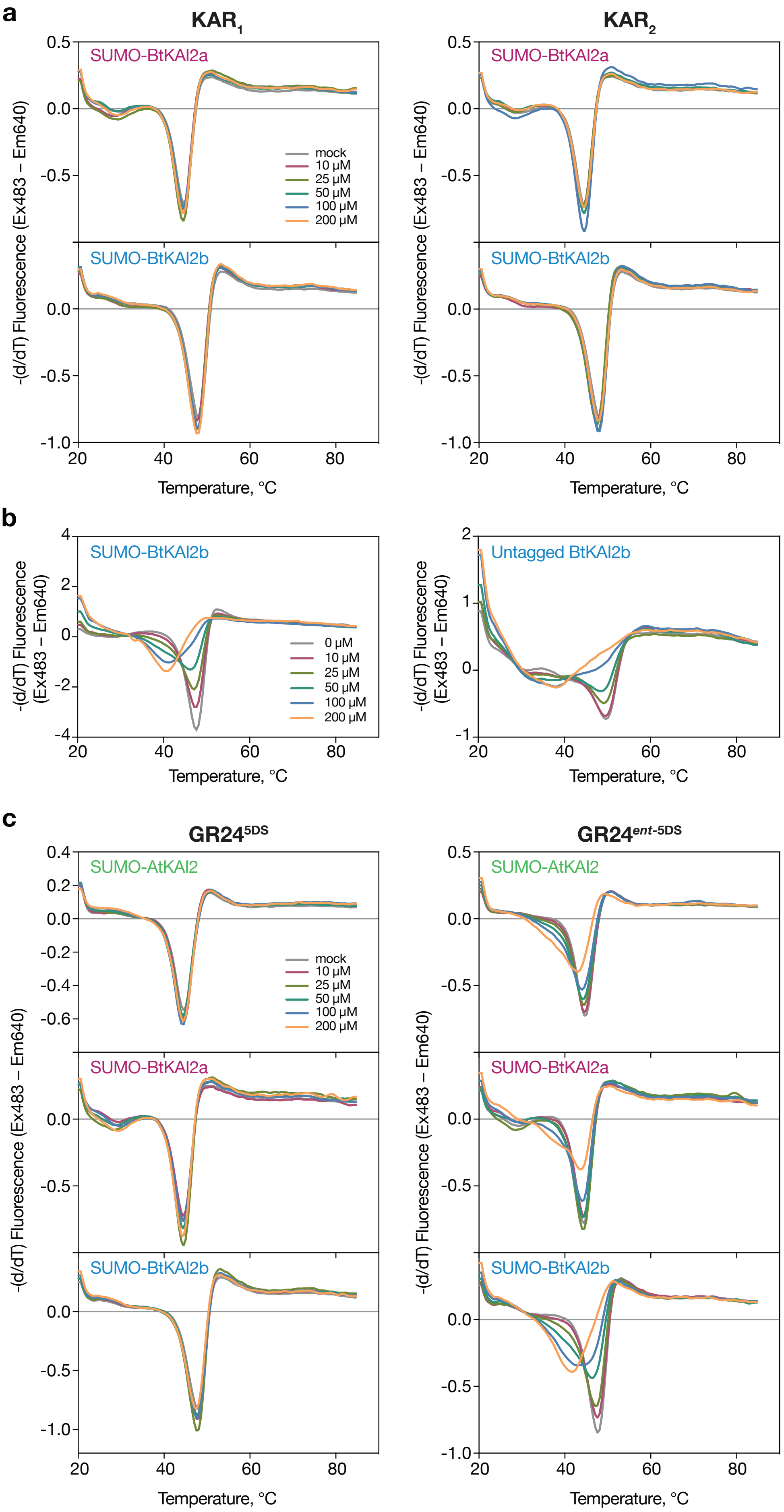

Supplementary Figure 9. BtKAI2a and BtKAI2b do not respond to karrikins in DSF assays

a, Differential scanning fluorimetry curves of SUMO-BtKAI2a and SUMO-BtKAI2b in presence of 0–200 µM KAR_1 or KAR_2. **b**, DSF responses of SUMO-tagged BtKAI2b (left) or untagged BtKAI2b (right) to 0–200 µM $GR24^{ent\text{-}5DS}$. c, DSF responses to 0–200 µM of the two enantiomers of GR24. Data are means of eight (a and c) or four (b) technical replicates at each concentration of ligand. Source data are provided as a Source Data file.

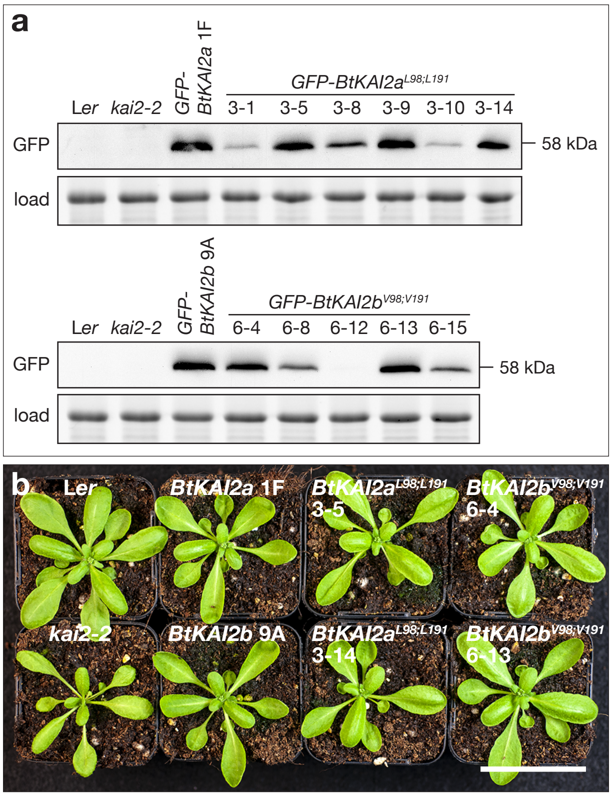

Supplementary Figure 10. Stable transgenic expression of BtKAI2 valine–leucine double exchange proteins in Arabidopsis

a, Immunoblots of total soluble protein extracted from 7-day-old seedlings of independent transgenic lines segregating in a 3:1 ratio for hygromycin resistance. Transgene expression in six lines expressing GFP-BtKAI2a$^{L98;L191}$ (upper panels) and five expressing GFP-BtKAI2b$^{V98;V191}$ (lower panels) were compared to a representative unmodified control (GFP-Bt-KAI2a 1F and GFP-BtKAI2b 9A respectively). Based on expression level two lines of each construct (3-5 and 3-14; 6-4 and 6-13) were selected and brought to homozygosity for further experiments. Protein blots were challenged with anti-GFP antibody. Equal gel loading was assessed by imaging total protein prior to blotting; the RbcL band is shown.
b, Rosette phenotypes of homozygous individuals expressing native and modified *GFP-BtKAI2* transgenes. Plants were 25 days old and grown under long day conditions as described in Methods. Scale bar: 50 mm. Source data are provided as a Source Data file.

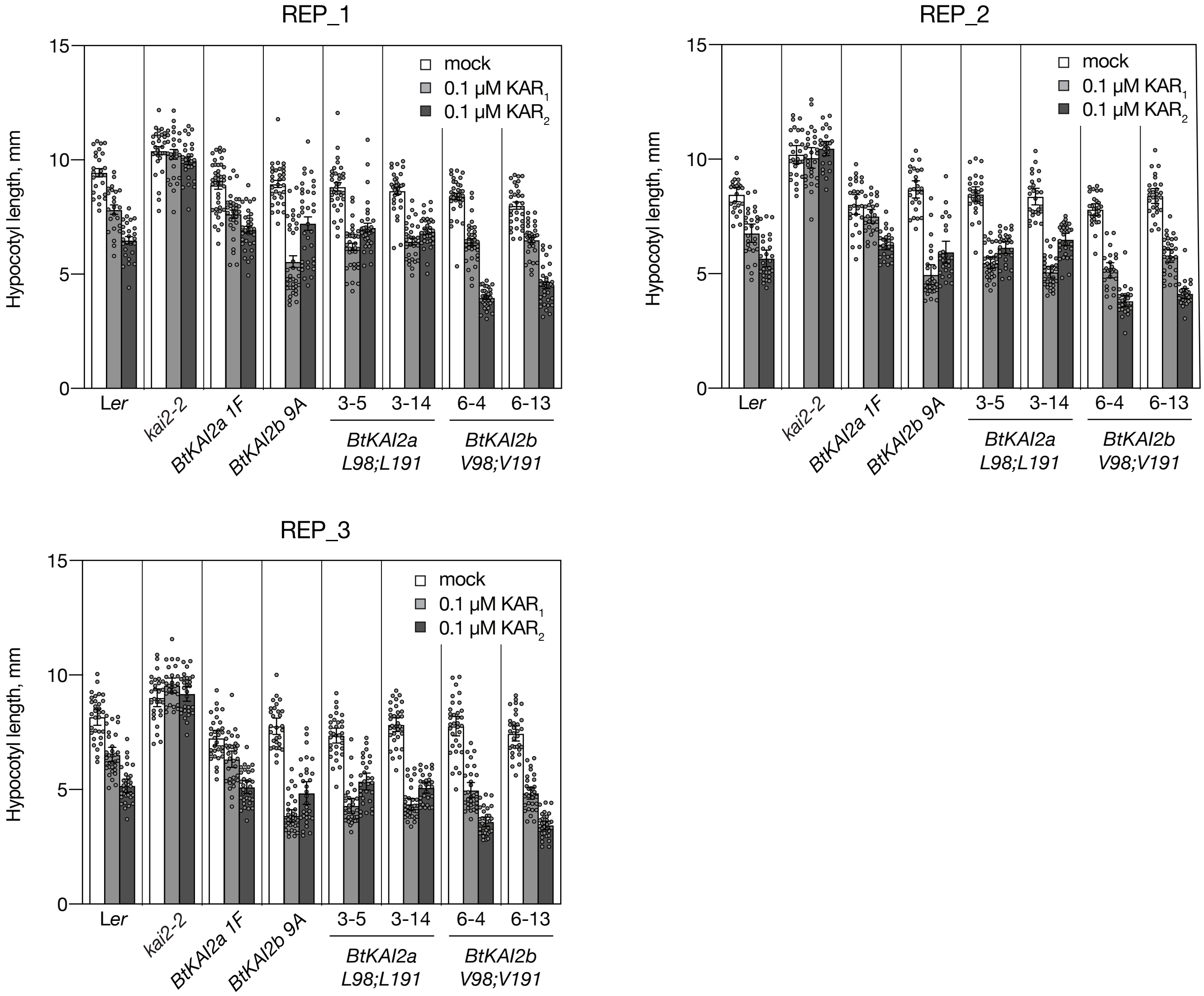

Supplemental Figure 11. Three experimental replicates of hypocotyl elongation assays with BtKAI2a and BtKAI2b transgenics (double exchange of residues 98 & 191)
Each panel depicts data from a separate experiment performed on the indicated date, which are shown in summarised format in Figure 4. Data are means ± SE, n=24 to 40 seedlings. Each dot corresponds to an individual seedling. Source data are provided as a Source Data file.

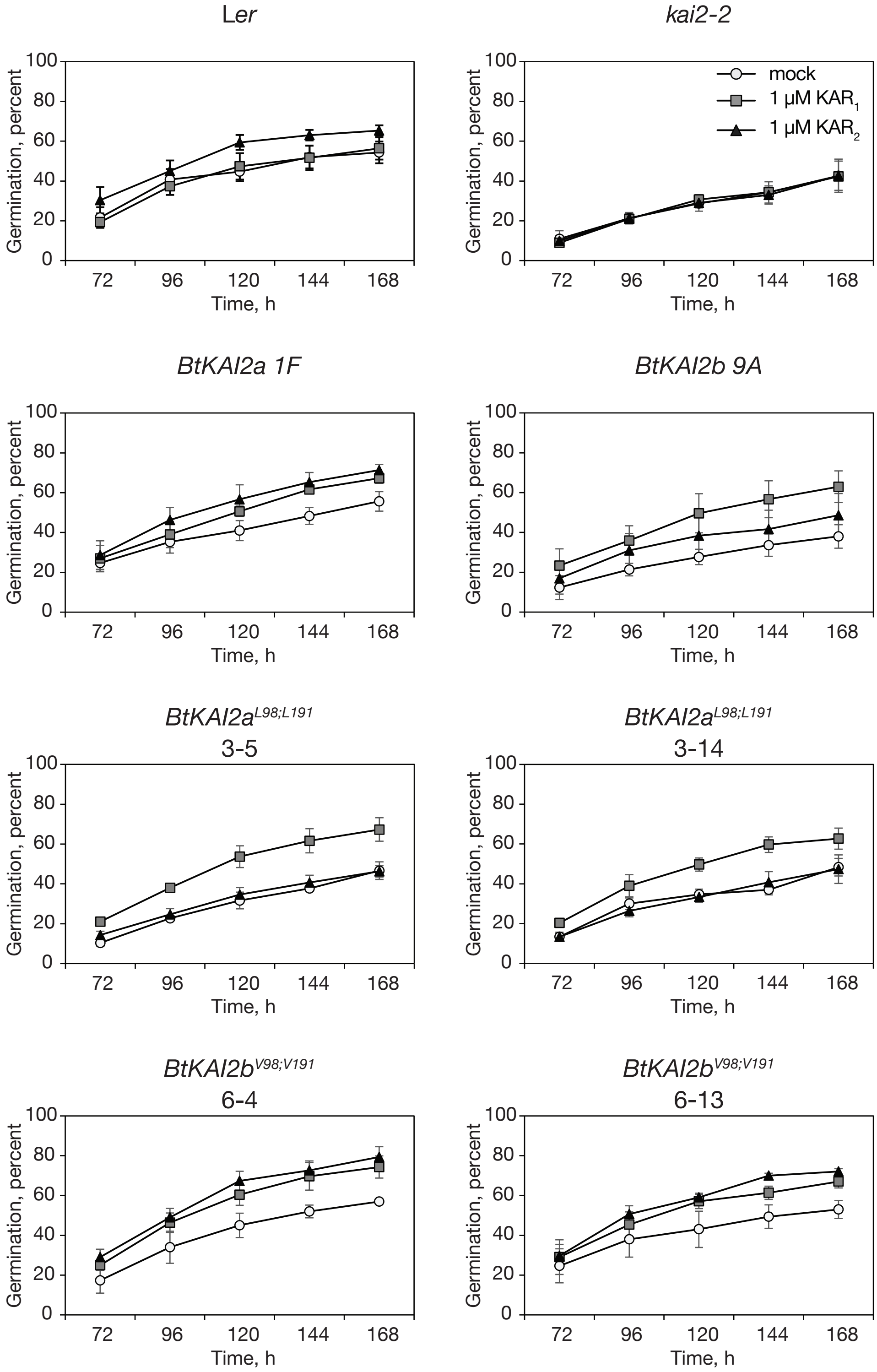

Supplementary Figure 12. Germination profiles of transgenic Arabidopsis seeds expressing BtKAI2 homologues (double exchange of residues 98 and 191)

Freshly harvested seeds (three batches per genotype, each batch harvested from three plants) were removed from freezer storage, surface-sterilised and sown on 0.7% agar supplemented with 0.02% acetone (mock), 1 µM KAR_1 or 1 µM KAR_2. Seeds were incubated under constant light at 25 °C. Seeds were examined for germination (radicle protrusion) 72 h after sowing and every 24 h thereafter. Data are means ± SE of three independent seed batches and 100 seed per batch. For the *BtKAI2a*$^{L98;L191}$ and *BtKAI2b*$^{V98;V191}$ transgenes, two independent, homozygous transgenic lines were analysed. Source data are provided as a Source Data file.

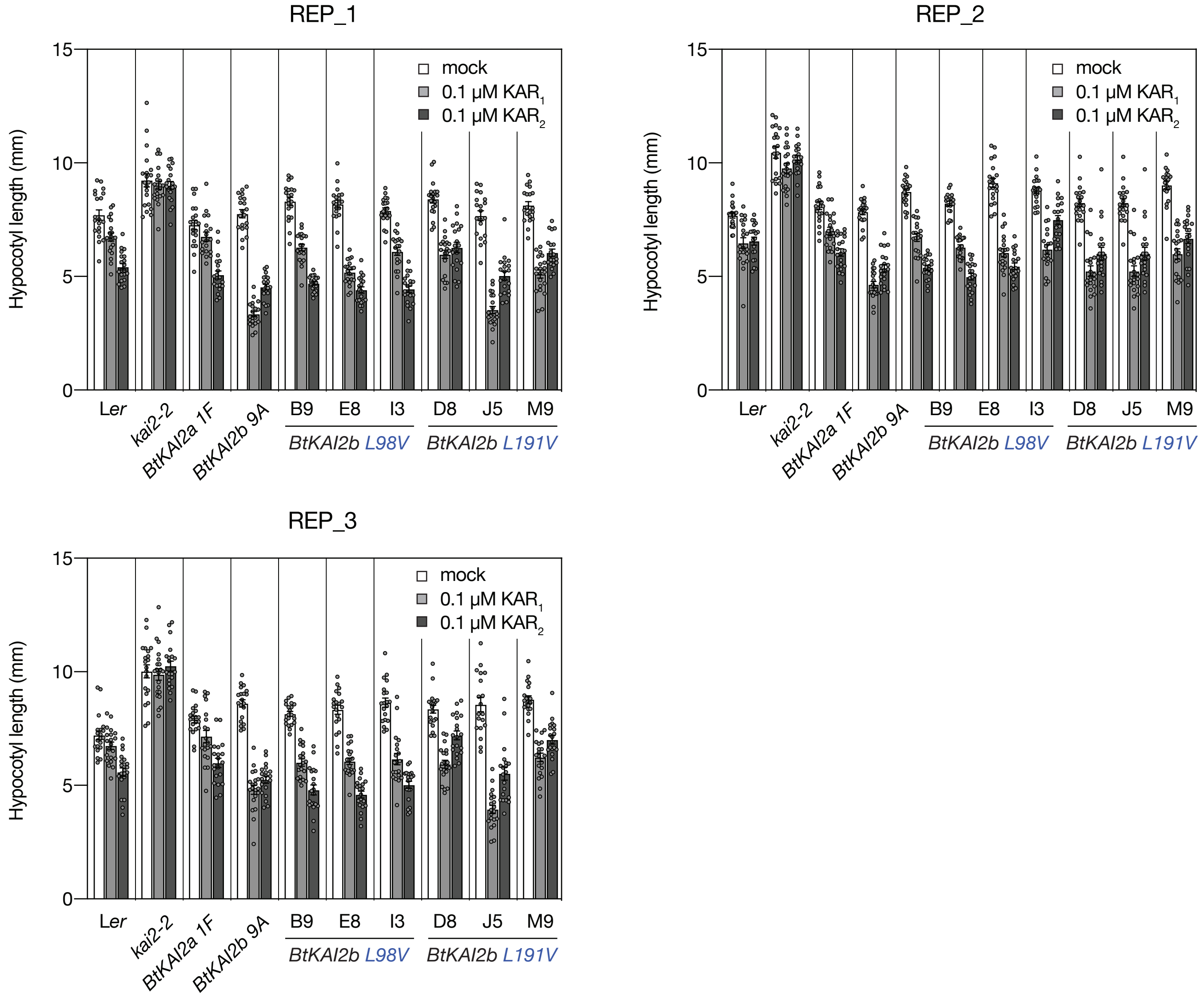

Supplemental Figure 13. Three experimental replicates of hypocotyl elongation assays with BtKAI2b transgenics (individual exchange of residues 98 & 191)

Each panel depicts data from a separate experiment performed on the indicated date, which are shown in summarised format in Figure 5. Data are means ± SE, n = 19-21 seedlings. Each dot corresponds to an individual seedling. Source data are provided as a Source Data file.

Supplementary Table 1. List of sequences identified in this study

KAI2

**Species**	**Gene name**	**Sequence ID**	**Source**	**Reference**
Arabidopsis lyrata	*AlKAI2*	AL7G13320.t1	Phytozome	This work
Arabidopsis thaliana	*AtKAI2*	At3g37470	TAIR	Waters et al (2012)
Brassica tournefortii (saharan mustard)	*BtKAI2a*	MG783328	GenBank/NCBI	This work
Brassica tournefortii (saharan mustard)	*BtKAI2b*	MG783329	GenBank/NCBI	This work
Brassica tournefortii (saharan mustard)	*BtKAI2c*	MG783330	GenBank/NCBI	This work
Brassica rapa (field mustard, AA genome)	*BrKAI2a*	XM_009111126.2	GenBank/NCBI	This work
Brassica rapa (field mustard, AA genome)	*BrKAI2b*	XM_009140247	GenBank/NCBI	This work
Brassica rapa (field mustard, AA genome)	*BrKAI2c*	XM_009144693	GenBank/NCBI	This work
Brassica nigra (black mustard, BB genome)	*BnKAI2a*	LFLV01000699.1	GenBank/NCBI	This work
Brassica nigra (black mustard, BB genome)	*BnKAI2b*	LFLV01001772.1	GenBank/NCBI	This work
Brassica oleracea (cabbage, CC genome)	*BoKAI2a*	XM_013766720.1	Phytozome	This work
Brassica oleracea (cabbage, CC genome)	*BoKAI2b*	XM_013738288.1	Phytozome	This work
Brassica oleracea (cabbage, CC genome)	*BoKAI2c*	XM_013778816.1	Phytozome	This work
Boechera stricta	*BsKAI2*	Bostr.30440s0001.1	Phytozome	This work
Capsella rubella	*CrKAI2*	Carubv10005485m	Phytozome	This work
Capsella grandiflora	*CgKAI2*	Cagra.1232s0005.1	Phytozome	This work
Eutrema salsugineum	*EsKAI2*	Thalv10025969m	Phytozome	Bythell-Douglas et al (2017)
Gossypium raymondii	*GrKAI2*	Gorai.003G028500.1	Phytozome	Bythell-Douglas et al (2017)
Prunus persica	*PpKAI2*	Ppa009957m	Phytozome	Bythell-Douglas et al (2017)
Brachypodium distachyon	*BdKAI2*	Bradi1g15880.1	Phytozome	Bythell-Douglas et al (2017)
Oryza sativa	*OsKAI2*	Os03g32270.1	Phytozome	Bythell-Douglas et al (2017)
Sorghum bicolor	*SbKAI2*	Sorbic.001G330000.1	Phytozome	Bythell-Douglas et al (2017)
Selaginella moellendorffii	*SmKAI2a*	Selmo_441991	Phytozome	Waters et al (2015)

D14

**Species**	**Gene name**	**Sequence ID**	**Source**	**Reference**
Arabidopsis lyrata	*AlD14*	AL3G13900.t1	Phytozome	This work
Arabidopsis thaliana	*AtD14*	AT3G03990.1	TAIR	Waters et al (2012)
Brassica oleracea (cabbage, CC genome)	*BoD14a*	LOC106343216	Phytozome	This work
Brassica oleracea (cabbage, CC genome)	*BoD14b*	LOC106343689	Phytozome	This work
Brassica nigra (black mustard, BB genome)	*BnD14a*	LFLV01002090	GenBank/NCBI	This work
Brassica nigra (black mustard, BB genome)	*BnD14b*	LFLV01000699	GenBank/NCBI	This work
Brassica rapa (field mustard, AA genome)	*BrD14a*	Brara.A03790	Phytozome	This work
Brassica rapa (field mustard, AA genome)	*BrD14b*	Brara.E03476	Phytozome	This work
B. tournefortii (saharan mustard)	*BtD14a*	MG783331	Phytozome	This work
B. tournefortii (saharan mustard)	*BtD14b*	MG783332	Phytozome	This work
Boechera stricta	*BsD14*	Bostr.2570s0310	Phytozome	This work
Capsella grandiflora	*CgD14*	Cagra.15970s0001	Phytozome	This work
Capsella rubella	*CrD14*	Carubv10014401m	Phytozome	This work
Eutrema salsugineum	*EsD14*	Thhalv10021292m	Phytozome	Bythell-Douglas et al (2017)
Gossypium raymondii	*GrD14*	Gorai.010G025600.1	Phytozome	Bythell-Douglas et al (2017)
Prunus persica	*PpD14*	ppa010005m	Phytozome	Bythell-Douglas et al (2017)
Brachypodium distachyon	*BdD14*	Bradi1g70930.3	Phytozome	Bythell-Douglas et al (2017)
Oryza sativa	*OsD14*	Os03g10620.1	Phytozome	Bythell-Douglas et al (2017)
Sorghum bicolor	*SbD14*	Sobic.001G465100.1	Phytozome	Bythell-Douglas et al (2017)

DLK2

**Species**	**Gene name**	**Sequence ID**	**Source**	**Reference**
B. tournefortii (saharan mustard)	*BtDLK2*	MG783333	GenBank/NCBI	This work

Supplementary Table 2. Analysis of BtKAI2c sequence using PROVEAN

Variant	BtKAI2c	Position	Consensus (Note 1)	Reverted (Note 2)	PROVEAN (Note 3)	cutoff = -2.5
1	R	36	K	K36R	-1.666	Neutral
2	R	98	V	V98R	-4.744	Deleterious
3	E	112	D	D112E	-0.937	Neutral
4	L	117	I	I117L	1.233	Neutral
5	E	129	D	D129E	-2.879	Deleterious
6	D	130	V	V130D	-0.063	Neutral
7	E	133	Q	Q133E	-0.112	Neutral
8	D	137	E	E137D	-2.17	Neutral
9	T	257	S	S257T	0.829	Neutral
10	I	263	L	L263I	-1.363	Neutral

Notes

1. Consensus is based on most common amino acid among KAI2 sequences from *Brassica* and *Arabidopsis.*
2. All variants were replaced with the consensus residue, and then resubmitted to PROVEAN with revertant mutations to recapitulate BtKAI2c sequence
3. This analysis only considers each substitution in turn, not the combined effect of multiple mutations

Supplementary Table 3. Oligonucleotides used in this study

Oligonucleotide	Sequence	Notes
**Cloning**		
BtD14a_F	AAAAAAGCAGGCTTCCATTATGAGTCAACACAACAT	BtD14a
BtD14a_R	CAAGAAAGCTGGGTTTTAAAGTCACCGAGGAAG	BtD14a
BtKAI2_universal_F	GGGGACAAGTTTGTACAAAAAAGCAGGCTTCATGGGAGGTGTGGTAGAGGA	All BtKAI2
RACE_R	GGGGACCACTTTGTACAAGAAAGCTGGGTCATTCTCAATAGACAATAGAC	BtKAI2a
Contig1_R	GGGGACCACTTTGTACAAGAAAGCTGGGTCCGAAACGACCCACTCACTTACC	BtKAI2b
Contig5_R	GGGGACCACTTTGTACAAGAAAGCTGGGTCCGAAGCAGAACCAAGCTAA	BtKAI2c
BtKAI2-SUMO-Gib_F	TGAGGCTCACCGCGAACAGATTGGAGGTATGGGAGGTGTGGTAGAGGAAG	pSUMO-BtKAI2a/b/c
BtKAI2a-SUMO-Gib_R	CGGATCTCAGTGGTGGTGGTGGTGGTGCTCAGACAGCGATGTCATTACGA	pSUMO-BtKAI2a
BtKAI2b-SUMO-Gib_R	CGGATCTCAGTGGTGGTGGTGGTGGTGCTCATACAGCAATGTCGTTGCGG	pSUMO-BtKAI2b
BtKAI2c-SUMO-Gib_R	CGGATCTCAGTGGTGGTGGTGGTGGTGCTCAGTAAGCAATGTCCTTGCG	pSUMO-BtKAI2b
BtKAI2b_L98V_F1	ATCTTTGTTGGTCACTCTGTCTCCGCCATG	For BtKAI2b(a) mutagenesis
BtKAI2b_L191V_R1	TTTGGAAAATGGTCTGAGCCACGGAGAGTG	For BtKAI2b(a) mutagenesis
BtKAI2b_L191V_F2	TCTCCGTGGCTCAGACCATTTTCCAAAGC	For BtKAI2b(a) mutagenesis
BtKAI2b_L98V_R2	CATGGCGGAGACAGAGTGACCAACAAAG	For BtKAI2b(a) mutagenesis
BtKAI2a_V98L_F1	ATCTTTGTTGGTCACTCTCTCTCCGCCATG	For BtKAI2a(b) mutagenesis
BtKAI2a_V191L_R1	TTTGGAAAATGGTCTGAGCCAGGGAGAGAG	For BtKAI2a(b) mutagenesis
BtKAI2a_V191L_F2	TCTCCCTGGCTCAGACCATTTTCCAAAG	For BtKAI2a(b) mutagenesis
BtKAI2a_V98L_R2	CATGGCGGAGAGAGAGTGACCAACAAAG	For BtKAI2a(b) mutagenesis
MW406	CACGAACTGACTAAGAGAGG	For cloning GFP-BtKAI2c cDNA
BK010	GAAACGATATACTCAGTACTTACCAC	For cloning GFP-BtKAI2c cDNA
BtKAI2c_R98V_F1	ACTCTGTTTCCGCCATGGTTG	For BtKAI2c mutagenesis
BtKAI2c_R98V_R1	GACCAACAAAGATACAAGACTCG	For BtKAI2c mutagenesis
BtKAI2c_E129D_D137E_F1	GACGTTGACTACCAAGGTGGTTTCGAACAAGAAGACCT	For BtKAI2c mutagenesis
BtKAI2c_E129D_D137E_R1	GTTCACGAATCTTGGGGAAGCAGAAACCATGAC	For BtKAI2c mutagenesis
AtKAI2_V98R_F1	CGTTCTGCCATGATTGGTGTC	For AtKAI2 mutagenesis
AtKAI2_V98R_R1	AGAGTGGCCAACAAAGATACAAG	For AtKAI2 mutagenesis
**Quantitative PCR**		
BtSTH7_qF	CATCTCCGGTTCTCTCTCACTTCT	Designed against At4g39070
BtSTH7_qR	CATTCTCTGCATAGTATTGCTCTGTC	Designed against At4g39070
BtDLK2_qF	GCTGCTTCTCCCAGGTATATAA	BtDLK2
BtDLK2_qR	GAAAGCAACCGCCCAAGCC	BtDLK2
AtDLK2_qF	GCTGCTTCTCCAAGGTATATAA	Designed against At3g24420
AtDLK2_qR	GAAATCAACCGCCCAAGCT	Designed against At3g24420
AtKUF1_qF1	AACCCGTCAGTCCGGTTTATGTG	Designed against At1g31350
AtKUF1_qR1	AACGACGGATGACGGTAAAGAATCC	Designed against At1g31350
BtKAI2_universal_qF	GGTCATCTTCCTCAGCTTAG	All BtKAI2
BtKAI2_a_qR1	CCATTAATTTAATTTAAAATCACTTCCC	BtKAI2a
BtKAI2_b_qR1	GCATTTAATTTCGCTAAAACTCTTG	BtKAI2b
BtKAI2_c_qR1	GAAACGATATACTCAGTACTTACCAC	BtKAI2c
CACS_qF	ACTCAGGAAGGTGTACGGTCA	Designed against At5g46630
CACS_qR	TGCATTTGGAACAGGTTTGT	Designed against At5g46630
BtCACS_qF	ACTCAGGAAGGTGTACGGTCA	Designed against At5g46630
BtCACS_qR	TGCATTTGGAACAGGTTTGT	Designed against At5g46630

SUPPLEMENTARY REFERENCES

1. Katoh K, Misawa K, Kuma Ki, Miyata T. MAFFT: a novel method for rapid multiple sequence alignment based on fast Fourier transform. Nucleic Acids Research 30, 3059-3066 (2002).

2. Kagiyama M, et al. Structures of D14 and D14L in the strigolactone and karrikin signaling pathways. Genes to Cells 18, 147-160 (2013).

3. Zhao L-H, et al. Crystal structures of two phytohormone signal-transducing α/β hydrolases: karrikin-signaling KAI2 and strigolactone-signaling DWARF14. Cell Res 23, 436 (2013).

4. Waters MT, Scaffidi A, Flematti G, Smith SM. Substrate-Induced Degradation of the α/β-Fold Hydrolase KARRIKIN INSENSITIVE2 Requires a Functional Catalytic Triad but Is Independent of MAX2. Molecular Plant 8, 814-817 (2015).
