# Supplementary material for: Divergent receptor proteins confer responses to different karrikins in two ephemeral weeds
Source: Nat Commun. 2020 Mar 9;11:1264. doi: 10.1038/s41467-020-14991-w (PMC7062792; doi:10.1038/s41467-020-14991-w)
Supplement: Supplementary file 3 — Description of Additional Supplementary Files [file 41467_2020_14991_MOESM3_ESM.pdf]

Description of Additional Supplementary Files

File Name: Supplementary Data 1
Description: Analysis of KAI2 homologues identified from The 1000 Plant Transcriptomes Project
