# Supplementary material for: Divergent receptor proteins confer responses to different karrikins in two ephemeral weeds
Source: Nat Commun. 2020 Mar 9;11:1264. doi: 10.1038/s41467-020-14991-w (PMC7062792; doi:10.1038/s41467-020-14991-w)
Supplement: Supplementary file 5 — Reporting Summary [file 41467_2020_14991_MOESM5_ESM.pdf]

nature research

Corresponding author(s): Mark Waters

Last updated by author(s): Jul 1, 2019

Reporting Summary

Nature Research wishes to improve the reproducibility of the work that we publish. This form provides structure for consistency and transparency in reporting. For further information on Nature Research policies, see Authors & Referees and the Editorial Policy Checklist .

Statistics

For all statistical analyses, confirm that the following items are present in the figure legend, table legend, main text, or Methods section.

n/a	Confirmed	
☐	☒	The exact sample size (*n*) for each experimental group/condition, given as a discrete number and unit of measurement
☐	☒	A statement on whether measurements were taken from distinct samples or whether the same sample was measured repeatedly
☐	☒	The statistical test(s) used AND whether they are one- or two-sided<br>*Only common tests should be described solely by name; describe more complex techniques in the Methods section.*
☒	☐	A description of all covariates tested
☐	☒	A description of any assumptions or corrections, such as tests of normality and adjustment for multiple comparisons
☐	☒	A full description of the statistical parameters including central tendency (e.g. means) or other basic estimates (e.g. regression coefficient) AND variation (e.g. standard deviation) or associated estimates of uncertainty (e.g. confidence intervals)
☐	☒	For null hypothesis testing, the test statistic (e.g. *F*, *t*, *r*) with confidence intervals, effect sizes, degrees of freedom and *P* value noted<br>*Give P values as exact values whenever suitable.*
☒	☐	For Bayesian analysis, information on the choice of priors and Markov chain Monte Carlo settings
☒	☐	For hierarchical and complex designs, identification of the appropriate level for tests and full reporting of outcomes
☒	☐	Estimates of effect sizes (e.g. Cohen's *d*, Pearson's *r*), indicating how they were calculated

Our web collection on statistics for biologists contains articles on many of the points above.

Software and code

Policy information about availability of computer code

Data collection	All software is publically available; no custom code was used in this study. Images of seedlings were analysed using ImageJ. Phylogenetic analysis used Geneious R10 software with the MAAFT alignments plug in (v1.37) and PhyML plugin (v2.2.3) as outlined in Methods. Protein homology modelling used PyMOL v1.3 and CASTp v3.0 as outlined in Methods. Quantitative PCR and DSF data collection was performed using Roche LightCycler 480 software v1.5.
Data analysis	GraphPad Prism for Mac v 8.0 was used for statistical analysis.

For manuscripts utilizing custom algorithms or software that are central to the research but not yet described in published literature, software must be made available to editors/reviewers. We strongly encourage code deposition in a community repository (e.g. GitHub). See the Nature Research guidelines for submitting code & software for further information.

Data

Policy information about availability of data

All manuscripts must include a data availability statement. This statement should provide the following information, where applicable:

- Accession codes, unique identifiers, or web links for publicly available datasets
- A list of figures that have associated raw data
- A description of any restrictions on data availability

Raw data (Excel files underlying charts, and uncropped images for photos and immunoblots) are available for all figures. The transcriptome datasets for Brassica tournefortii generated in this study are available in the NCBI SRA database (https://www.ncbi.nlm.nih.gov/sra) under accession SRP128835. Accession numbers for sequences used in phylogeny reconstruction are provided in supplementary data. All other datasets are available from the corresponding author upon reasonable request.

Field-specific reporting

Please select the one below that is the best fit for your research. If you are not sure, read the appropriate sections before making your selection.

- [x] Life sciences
- [ ] Behavioural & social sciences
- [ ] Ecological, evolutionary & environmental sciences

For a reference copy of the document with all sections, see nature.com/documents/nr-reporting-summary-flat.pdf

Life sciences study design

All studies must disclose on these points even when the disclosure is negative.

Sample size	Samples for gene expression analyses were selected based on standard minimal sizes for this type of experiment, bearing in mind the complexity of multiple genotypes and multiple treatments (n = 3 biological replicates). For measurements of seedling hypocotyl growth, greater than 12 (typically 20) individual seedlings were measured, in each of three experimental replicates; thus there were n=3 samples of 12-20 seedlings as described in individual figure legends. For seed germination experiments, at least three batches of seed, each harvested from different groups of 3-4 individual plants, were tested in parallel; data were thus collected from n=3 batches of 75 seed for each genotype/treatment combination, with two independent transgenic lines where appropriate.
Data exclusions	None
Replication	Experiments were performed at least twice where relevant (e.g. gene expression analyses). For hypocotyl elongation measurements, the figures represent three experimental replicates, with two to three independent transgenic lines where appropriate. For seed germination data, the experiments were performed on two completely separate sets of seed batches; data presented are from one set of three batches as described above.
Randomization	Randomisation was not a major feature of our study, with the exception of growth of plants for seed germination experiments, where plants were assigned random positions in trays using Excel to generate a randomised order. In addition, petri dishes containing seed for germination counting or for hypocotyl growth were randomly mixed by hand prior to placing in the incubators.
Blinding	Investigators were not routinely blinded, as much of the work did not require subjective assessment. However, for seed germination assays, genotypes were assigned a random code by another investigator in the lab, and deconvoluted after measurements were complete.

Reporting for specific materials, systems and methods

We require information from authors about some types of materials, experimental systems and methods used in many studies. Here, indicate whether each material, system or method listed is relevant to your study. If you are not sure if a list item applies to your research, read the appropriate section before selecting a response.

Materials & experimental systems

n/a	Involved in the study	
☐	☒	Antibodies
☒	☐	Eukaryotic cell lines
☒	☐	Palaeontology
☒	☐	Animals and other organisms
☒	☐	Human research participants
☒	☐	Clinical data

Methods

n/a	Involved in the study	
☒	☐	ChIP-seq
☒	☐	Flow cytometry
☒	☐	MRI-based neuroimaging

Antibodies

Antibodies used	anti-GFP (rabbit polyclonal/commercial), ThermoFisher A11122; anti-KAI2 (rabbit polyclonal/custom made), Waters et al Mol Plant 2015; anti-actin (mouse monoclonal/commercial), Sigma A0480; anti-c-myc (mouse monoclonal/commercial) Genscript A00704.
Validation	anti-GFP: refer https://www.thermofisher.com/antibody/product/GFP-Tag-Antibody-Polyclonal/A-11122 and references therein<br>anti-KAI2: refer Waters et al. (2015) Mol Plant 8, 814-817<br>anti-actin: refer https://www.sigmaaldrich.com/catalog/product/sigma/a0480?lang=en&region=AU and references therein
